# Single-step doxorubicin-selected cancer cells overexpress the ABCG2 drug transporter through epigenetic changes

**DOI:** 10.1038/sj.bjc.6604334

**Published:** 2008-04-01

**Authors:** A M Calcagno, J M Fostel, K K W To, C D Salcido, S E Martin, K J Chewning, C-P Wu, L Varticovski, S E Bates, N J Caplen, S V Ambudkar

**Affiliations:** 1 Laboratory of Cell Biology, Center for Cancer Research, National Cancer Institute, NIH, DHHS, Bethesda, MD 20892, USA; 2 Alpha-Gamma Technologies Inc., Raleigh, NC 27609, USA; 3 Medical Oncology Branch, Center for Cancer Research, National Cancer Institute, NIH, DHHS, Bethesda, MD 20892, USA; 4 Laboratory of Human Carcinogenesis, Center for Cancer Research, National Cancer Institute, NIH, DHHS, Bethesda, MD 20892, USA; 5 Gene Silencing Section, Genetics Branch, Center for Cancer Research, National Cancer Institute, NIH, DHHS, Bethesda, MD 20892, USA

**Keywords:** multidrug resistance (MDR), doxorubicin, ABCG2, epigenetics, single-step selection

## Abstract

Understanding the mechanisms of multidrug resistance (MDR) could improve clinical drug efficacy. Multidrug resistance is associated with ATP binding cassette (ABC) transporters, but the factors that regulate their expression at clinically relevant drug concentrations are poorly understood. We report that a single-step selection with low doses of anti-cancer agents, similar to concentrations reported *in vivo,* induces MDR that is mediated exclusively by ABCG2. We selected breast, ovarian and colon cancer cells (MCF-7, IGROV-1 and S-1) after exposure to 14 or 21 nM doxorubicin for only 10 days. We found that these cells overexpress ABCG2 at the mRNA and protein levels. RNA interference analysis confirmed that ABCG2 confers drug resistance. Furthermore, *ABCG*2 upregulation was facilitated by histone hyperacetylation due to weaker histone deacetylase 1-promoter association, indicating that these epigenetic changes elicit changes in *ABCG*2 gene expression. These studies indicate that the MDR phenotype arises following low-dose, single-step exposure to doxorubicin, and further suggest that ABCG2 may mediate early stages of MDR development. This is the first report to our knowledge of single-step, low-dose selection leading to overexpression of *ABCG*2 by epigenetic changes in multiple cancer cell lines.

Cancer remains a major health issue and is responsible for one in four deaths in the United States (Zhou *et al*, 2001; Jemal *et al*, 2006). The development of multidrug resistance (MDR) to chemotherapeutic agents plays a major role in the failure of cancer therapy. Resistance to therapy can result from decreased drug uptake, increased DNA repair or drug inactivation (Gottesman *et al*, 2002). However, the most common mechanism is the overexpression of ATP binding cassette (ABC) drug transporters that protect cells by increasing drug efflux. Several investigators have reported increased ABC transporter expression following drug treatment (reviewed in Lage, 2003). However, a debate surrounds the relevance of multistep and continuous selections with clinically unattainable concentrations commonly used to study cancer drug resistance.

ATP binding cassette transporters are a large superfamily of proteins expressed in normal cells in varying amounts. Some members of this superfamily function to eliminate endogenous and xenobiotic metabolites, and over 12 ABC transporters have been linked to MDR. Importantly, there is a substantial overlap in the list of substrates for ABCB1, ABCG2 and the various ABCC family members linked with MDR, in spite of significant differences in the primary sequences of these transporters (Haimeur *et al*, 2004). This phenomenon makes treatment of multidrug-resistant cancer unsuccessful despite the various chemotherapeutic drugs that are available.

Cell-based studies of MDR to date have involved establishing multidrug-resistant cancer cell lines through continual or multistep drug selection and using high drug concentrations. Although these cell lines provided an important tool for understanding ABC transporter function, these selection regimens do not mimic *in vivo* drug concentrations or dosing frequencies that occur in cancer patients. In this study, we used short, low-dose drug selection of cancer cell lines to more closely simulate *in vivo* drug concentrations such as those used in the liposomal doxorubicin formulation, which is commonly used as a single-agent therapy for metastatic breast cancer patients with greater cardiac risks (Lorusso *et al*, 2007) and metastatic ovarian cancer refractory to paclitaxel- and platinum-based chemotherapy (Thigpen *et al*, 2005). Liposomal doxorubicin is known to have a slow and sustained release of drug from the liposomal carrier with a half-life of 55 h (Allen *et al*, 2006). Our *in vitro* studies utilised doses significantly below the IC_50_ of doxorubicin for MCF-7 cells; concentrations were kept constant over a 10-day period to mimic the release characteristics from a liposomal carrier. In contrast to multistep exposure to doxorubicin, where either ABCB1 or ABCC1 is the dominant ABC transporter causing MDR (Shen *et al*, 1986; Barrand *et al*, 1994; Mehta, 1994), we show here that following single-step selection other transporters, including ABCC4 and ABCG2, are overexpressed. Importantly, ABCG2 was found to confer resistance to doxorubicin in breast, ovarian and colon cancer cell lines. Sequencing of ABCG2 in the single-step-selected MCF-7 sublines revealed that our *in vitro* selection resulted in overexpression of the wild-type ABCG2 and not the gain-of-function mutations either G or T at amino acid 482. While other investigators have reported higher resistance to anthracycline with the 482 mutants (Honjo *et al*, 2001), we also report increased resistance to doxorubicin in the sublines with wild-type ABCG2 compared to the parental MCF-7 cells in our studies. Overexpression of ABCG2 was also observed following single-step low-dose selection with etoposide. This study shows that ABCG2 may play a critical role in the development of drug resistance.

## MATERIALS AND METHODS

### Cell culture

The MCF-7 breast cancer cell line and the multistep doxorubicin-selected subline MCF7/ADR were a gift of Dr Kapil Mehta (MD Anderson Cancer Center, Houston, TX, USA) (Mehta, 1994). The multistep doxorubicin-selected MCF-7 cells were cultured in high-dose doxorubicin (860 nM) every alternate passage as previously described (Mehta, 1994). The generation of MCF-7/ADR-VP3000 and MCF7/FLV1000 cell lines by multistep selection was described previously (Chen *et al*, 1990; Robey *et al*, 2001). Spectral karyotyping of these cells matched those described by others (Kytola *et al*, 2000). Parental HEK293 cells (293 human embryonic kidney cells) and the MRP4-overexpressing HEK293/4.63 cells were gifts of P Borst (The Netherlands Cancer Institute, Amsterdam, The Netherlands) (Wielinga *et al*, 2002). HEK293/4.63 cells were reported to express significantly more MRP4 (Reid *et al*, 2003) without overexpression of other ABC drug transporters and were cultured as previously described (Wu *et al*, 2005). The ovarian IGROV-1 cells were obtained from the Developmental Therapeutics Program, NCI. S-1 cells, cloned from LS174T colon carcinoma cells, were described previously (Rabindran *et al*, 1998).

### Drug selection protocol

Drug-resistant MCF-7, IGROV-1 and S-1 clones were established, employing a single-step selection with either 14 or 21 nM doxorubicin or 300 nM etoposide treatment for 10 days followed by culturing in drug-free medium. Briefly, 10 000 cells were seeded in a 100 × 20 mm tissue culture dish with drug. Medium with drug was changed three times during the selection and, subsequently, the cells were maintained in medium without drug. Individual cells were selected randomly using sterile cloning disks pretreated in trypsin and placed in separate wells within a 24-well plate. Clones were grown to confluency and expanded in drug-free media. Expanded clones were retested for drug resistance before any further studies. Detailed characterisation of all clones began approximately 8–12 weeks after single-step selection. The resistant phenotype in isolated clones was examined 24 weeks after initial treatment and was found to be stable.

### RNA isolation and quantitative RT–PCR

RNA was isolated from cells grown in six-well plates to characterise ABC transporter expression in all cell lines as described previously (Wu *et al*, 2005). Real-time quantitative RT–PCR (qRT–PCR) was performed using the LightCycler RNA Master SYBR Green kit and the LightCycler 480 (Roche Biochemicals, Indianapolis, IN, USA). Plasma membrane calcium ATPase 4 (*PMCA4*) was used as the reference gene (Calcagno *et al*, 2006). Specific PCR conditions and primer sequences for genes are described in Supplementary Information.

### Western blotting

For western blotting assays, equivalent numbers of cells were harvested and lysed as described previously (Wu *et al*, 2005). Nitrocellulose membranes were probed with the appropriate primary antibodies specific to the protein of interest. The following antibodies were used: C219 (1 : 2000), ABCB1 (Kartner *et al*, 1985); M2 III-6 (1 : 50) (Alexis Biochemical, Lausen, Switzerland), ABCC2 (Paulusma *et al*, 1996); anti-BCRP (1 : 1000) (Kamiya Biochemical, Seattle, WA, USA), ABCG2 (Maliepaard *et al*, 2001); and M4 1-80 (1 : 200) (Kamiya Biochemical), ABCC4 (Leggas *et al*, 2004). Glyceraldehyde-3-phosphate dehydrogenase (GAPDH; 1 *μ*g ml^−1^) (Zymed Laboratories, San Francisco, CA, USA) was used as a loading control.

### Confocal imaging

MCF-7 parental cells and 21 nM single-step-selected cells were seeded at 10 000 cells per well on Lab-Tek chamber slides with covers (Nunc, Rochester, NY, USA). After 48 h, the chambers were washed twice with PBS (Gibco-Invitrogen, Carlsbad, CA, USA), fixed (IntraPrep kit, Beckman Coulter, Fullerton, CA, USA) for 15 min at room temperature (RT), washed three times with wash buffer (PBS/0.1% bovine serum albumin) and blocked for 1 h at RT with wash buffer. Following two washes with PBS, a 1 : 100 solution of the 5D3 clone of the mouse anti-human ABCG2 antibody (eBioscience, San Diego, CA, USA) in wash buffer was added to all cell chambers for 1 h at RT followed by three washes with wash buffer. The anti-mouse Alexa Fluor 488-conjugated antibody (Invitrogen, Carlsbad, CA, USA) (diluted 1 : 250 in wash buffer) was incubated with the cells for 30 min at RT. Following two washes with wash buffer, the chambers and seal were removed. Slides were air dried and mounted in aqueous mounting media with 4′,6-diamidino-2-phenylindole (DAPI) (Vector Laboratories Inc., Burlingame, CA, USA). Images were acquired using an LSM 510 confocal system mounted on a Zeiss Axiovert 100M microscope with an oil immersion Plan-Apochromat × 63/1.4 NA DIC objective lens (Carl Zeiss, Thornwood, NY, USA). Excitation of Alexa Fluor 488 was performed using the 488 nm line from a 25 mW argon laser while excitation of DAPI was performed by using the 364 nm line from an 80 mW UV laser. The Alexa Fluor 488 emission was collected using a long-pass 505 nm filter and the DAPI emission was collected using a band pass 390–465 nm filter.

### Cytotoxicity assay

Sensitivities of cell lines to various chemicals were examined using the Cell-Counting Kit (CCK) technique as detailed previously (Ishiyama *et al*, 1996). Cells were plated at a density of 1000–5000 cells per well in 96-well plates containing 100 *μ*l of culture medium. After 24 h incubation at 37°C, drugs were added into wells to a final volume of 200 *μ*l per well and incubated for an additional 72 h. CCK reagent was then added into each well and incubated for 4 h before reading at a wavelength of 450 nm. IC_50_ values were calculated from dose–response curves obtained from at least three independent experiments.

### siRNA design and silencing of *ABCG2* expression

The siRNAs employed were designed and synthesised by Qiagen Inc. (Germantown, MD, USA). The ABCG2-2 siRNA duplex (siG2-2) consisted of 5′-GGAUAAGCCACUCAUAGAAdtdT (sense) and 5′-UUCUAUGAGUGGCUUAUCCdTdG (antisense) strands. The negative siRNA duplex (siNeg) consisted of 5′-ACGUGACACGUUCGGAGAAdTdT and 5′-UUCUCCGAACGUGUCACGUdTdT strands. For siRNA transfections, siRNA (1.25 or 5 pmol) was added to individual wells of a 96-well plate in 25 *μ*l serum-free DMEM. Oligofectamine (0.7 *μ*l; Invitrogen) was subsequently added to siRNA-containing wells in 25 *μ*l serum-free DMEM to give a final lipid/siRNA ratio of 2 : 1 (w/w). The resulting mixture was allowed to complex for 30 min at ambient temperature. Cells (5000 cells per well – RNA analysis; 1000 cells per well – cytotoxicity assay) were added in 50 *μ*l DMEM supplemented with 20% FBS to yield transfection mixtures of 12.5 or 50 nM siRNA in DMEM containing 10% FBS. This final mixture was incubated at ambient temperature for 45 min before being placed at 37°C in a humidified atmosphere containing 5% CO_2_. For initial screening of siRNA and protein analysis, the transfection protocol was performed as described above using six-well plates and the reagents were scaled up 30-fold using 50 nM siRNA. *ABCG2* mRNA levels were analysed 48 h after transfection by real-time RT–PCR for the initial siRNA screening. RNA levels for the comparison of 12.5 and 50 nM were analysed 48 h after transfection using the QuantiGene Reagent System (Panomics, Fremont, CA, USA) and normalised to cyclophilin B (*PPIB*) mRNA. The results reflect the average and s.d. of at least five replicate experiments. ABCG2 protein levels were measured by western blotting as described above 48 h after siRNA transfection. For drug analysis, cells were grown for 48 h before adding various concentrations of mitoxantrone for 72 h as described above.

### Chromatin immunoprecipitation assay

Chromatin immunoprecipitation (ChIP) assays were performed on MCF-7, MCF7/FLV1000 and five single-step doxorubicin-selected clones. Briefly, cells (1 × 10^6^) were crosslinked with 1% formaldehyde for 10 min at 37°C, quenched with 0.125 M glycine for 5 min at RT and rinsed in ice-cold PBS containing 5 mM sodium butyrate (Sigma, St Louis, MI, USA). Cells were scraped and re-suspended in a lysis buffer (Active Motif, Carlsbad, CA, USA) with the addition of complete protease inhibitor cocktail (Roche Applied Science, Indianapolis, IN, USA). DNA–protein complexes were sheared using an enzymatic shearing kit (Active Motif) to yield DNA fragments below 800 bp, as determined by agarose gel electrophoresis. Chromatin immunoprecipitations were carried out overnight at 4°C with 10 *μ*g of anti-acetylated H3 (AcH3), 5 *μ*g of anti-histone deacetylase 1 (HDAC1; Upstate Biotechnology, Charlottesville, VA, USA) and 2 *μ*g of anti-RNA polymerase II (Pol II; Santa Cruz Biotechnology, Santa Cruz, CA, USA). Primer sets encompassing approximately 1.6 kb of the *ABCG*2 promoter were used to map changes in histone acetylation after drug selection. The amount of immunoprecipitated DNA was assessed by quantitative PCR, using primers spanning the proximal (−293 to –139 nt) and distal (−1527 to –1268 nt) promoter region of *ABCG2*, and compared to the amount of input DNA prior to immunoprecipitation. The numbering of the nucleotides was assigned relative to the transcriptional start site designated in GenBank sequence AF151530. Amplification of immunoprecipitated DNA was achieved using previously described methods (To *et al*, 2006).

## RESULTS

### Single-step doxorubicin selection results in ABCG2 overexpression

**Figure 1:**
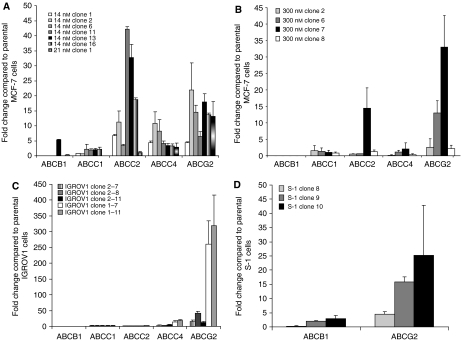
Single-step drug-selected clones overexpress *ABCG*2 mRNA. (**A**) Characterisation of selected ABC transporter gene expression levels in several single-step doxorubicin-resistant MCF-7 clones, described in Materials and Methods. The average fold change compared to parental MCF-7 cells±s.d. (*n*=4) was calculated using delta delta *C*_t_ method from real-time RT–PCR data. The colour-coded key for the clones is given in the figure. (**B**) Single-step etoposide-resistant MCF-7 clones are described in Materials and Methods. The average fold change compared to parental MCF-7 cells±s.d. (*n*=4) was calculated using delta delta *C*_t_ method from real-time RT–PCR data. The colour-coded key for the clones is given in the figure. (**C**) Doxorubicin-resistant IGROV-1 clones were established employing a single-step selection with 14 nM doxorubicin treatment as described previously. The average fold change compared to parental IGROV-1 cells±s.d. (*n*=4) was calculated using delta delta *C*_t_ method from real-time RT–PCR data. The colour-coded key for the clones is given in the figure. (**D**) Doxorubicin-resistant S-1 clones were established employing a single-step selection with 21 nM doxorubicin. The average fold change compared to parental S-1 cells±s.d. (*n*=4) was calculated using delta delta *C*_t_ method from real-time RT–PCR data. The key for the clones is given in each panel.

**Figure 2:**
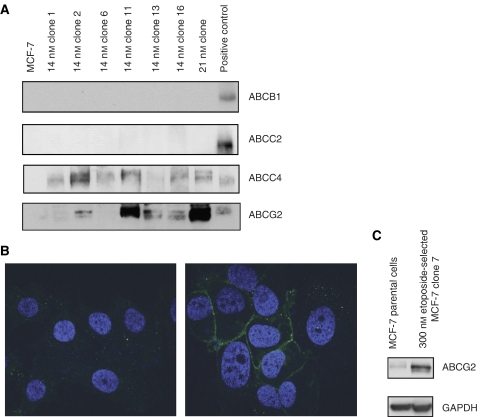
Single-step drug-selected MCF-7 clones overexpress ABCG2 protein. (**A**) Western blotting analysis of selected ABC drug transporters in doxorubicin-selected MCF-7 clones. Lanes 1–8 are the same in all panels and only lane 9 varies in each panel. A total of 100 000 cells were loaded per lane for lanes 1–8. Lane 1, parental MCF-7; lane 2, 14 nM clone 1; lane 3, 14 nM clone 2; lane 4, 14 nM clone 6; lane 5, 14 nM clone 11; lane 6, 14 nM clone 13; lane 7, 14 nM clone 16; and lane 8, 21 nM clone. Top panel lane 9, 10 000 MCF7/ADR cells (positive control); second panel lane 9, 20 000 LLCPK-ABCC2 cells (positive control); third panel lane 9, 2000 ABCC4-HEK293 cells (positive control); and bottom panel lane 9, 5000 MCF7/FLV1000 cells (positive control). Immunoblotting with antibodies specific for each transporter (as indicated on the right side of each panel) was carried out as described in Materials and Methods. (**B**) Cell surface localisation of ABCG2 in 21 nM doxorubicin-selected MCF-7 clone. 5D3 binding in MCF-7 (left panel) and 21 nM clone cells (right panel) is shown. Confocal imaging was performed on permeabilised cells following binding with the ABCG2-specific 5D3 antibody (Zhou *et al*, 2001) as described in Materials and Methods. Nuclei were stained with DAPI, blue stain (showing as light grey in print); Alexa-Fluor 488 secondary antibody (green) (white in print) was used to localise 5D3 binding. (**C**) Western blotting analysis of ABCG2. A total of 100 000 cells were loaded per lane for lanes 1 and 2. Lane 1, MCF-7; lane 2, 300 nM etoposide-selected MCF-7 clone 7. Glyceraldehyde-3-phosphate dehydrogenase was used as a loading control.

The gene expression profiles of select ABC transporters (ABCB1, ABCC1, ABCC2, ABCC4 and ABCG2) linked to MDR (Perez-Tomas, 2006) were assayed using qRT–PCR in MCF-7 cells subjected to drug selection. These transporters have been associated with the MDR phenotype in various cell lines (Perez-Tomas, 2006). Briefly, ten thousand cells (in a 100 × 20 mm dish) were treated for 10 days with low-dose doxorubicin. Drug selection with either 14 or 21 nM doxorubicin was performed. These doses were chosen because they are significantly below the IC_50_ of doxorubicin for MCF-7 cells, which is ∼90 nM (Table 1). Following the 10-day drug selection, single isolated cells were chosen randomly for expansion. The ABC transporter expression patterns for six doxorubicin-resistant MCF-7 clones selected with 14 nM doxorubicin and one 21 nM doxorubicin-resistant clone are shown in Figure 1A. We also performed selections at higher concentrations of doxorubicin (70, 140 and 210 nM). No MCF-7 cells survived this selection pressure, suggesting that pre-existing cells exhibiting high levels of ABC transporters within the MCF-7 parental population were not present. In contrast to the previously established multistep-selected line MCF7/ADR (Mehta, 1994), most of the low-concentration clones did not express *ABCB*1 (Figure 1A). Only the 14 nM doxorubicin-selected MCF-7 clone 13 showed a slight increase in ABCB1 at the mRNA level; yet, no detectable levels of ABCB1 protein were found in this clone (Figure 2A). On the contrary, all single-step-selected clones showed overexpression of *ABCG*2 (4- to 30-fold) when compared to their parental MCF-7 cells.

To determine if this pattern of expression of MDR-associated ABC transporters following below IC_50_ selection was drug and cell line independent, we selected MCF-7 cells with a single-step selection using 300 nM etoposide and two additional cancer lines, IGROV-1 ovarian cancer cells and S-1 colon tumor cells, with 14 and 21 nM doxorubicin, respectively (Figure 1B–D). With lower etoposide concentrations of 50, 100 and 200 nM, all MCF-7 parental cells were able to survive the selection pressure. For IGROV-1 cells, no parental cells survived the 21 nM selection. We also studied five sublines derived from IGROV-1 cells obtained using 14 nm doxorubicin as well as S-1-resistant clones, obtained at a 21 nM concentration of doxorubicin. In all cases, *ABCG2* was the dominant overexpressed gene; the etoposide-selected MCF-7 cells and the doxorubicin-selected S-1 increased *ABCG*2 mRNA levels 43-fold while IGROV-1 cells exhibited a nearly 400-fold increase. This suggests that *ABCG2* overexpression is an early molecular marker of the development of MDR in these three cancer cell types. Two other ABC transporters *ABCC*2 and *ABCC*4 were also overexpressed in some sublines of MCF-7 cells selected with doxorubicin, exhibiting a 2- to 24-fold and 3- to 10-fold overexpression of mRNA when compared to the parental MCF-7 cells, respectively.

To examine if the protein expression of these transporters was also changed, several immunoblots were performed. Consistent with the mRNA analysis, neither the parental cell line nor any of the single-step doxorubicin-resistant MCF-7 clones including clone 13, which showed a slight increase in ABCB1 mRNA, expressed ABCB1 protein at detectable levels whereas the MCF7/ADR cells expressed high levels of ABCB1 (Figure 2A, top panel). Surprisingly, no ABCC2 protein was detected in any of the MCF-7 sublines, although several clones showed a 2- to 24-fold increase of mRNA compared to the parental MCF-7 cells (Figure 2A, second panel). The reason for the lack of correlation is not yet clear. ABCC4 protein was detected in all clones examined (Figure 2A, third panel). Five of these doxorubicin-selected MCF-7 clones showed enhanced levels of ABCG2 protein compared to parental MCF-7 cells, with two clones, 14 nM clone 11 and 21 nM clone, showing very high levels (Figure 2A, bottom panel). This analysis showed enhanced mRNA and protein levels, which was consistent with the increase in the function and cell surface expression of ABCG2 in these MCF-7 single-step clones. Interestingly, overexpression of ABCG2 was retained in the absence of long-term selection, as both ABCG2 mRNA and protein levels were found to be increased in cells grown for 24 weeks in the absence of doxorubicin following the initial drug treatment (data not shown).

To further support these western blotting results, which showed that the ABCG2 protein was found in the single-step clones, confocal microscopy studies were also performed to determine if the ABCG2 protein in the single-step-selected cells was properly localised to the plasma membrane. Using the 5D3 antibody, which detects an extracellular epitope of ABCG2 (Zhou *et al*, 2001), the cell surface expression of ABCG2 was detected in non-permeabilised cells. The MCF-7 parental cells did not show binding of 5D3 (Figure 2B, left panel); however, the 21 nM clone showed a distinct pattern of binding of 5D3 (Figure 2B, right panel) in intact cells. This suggested that ABCG2 predominately localised to the plasma membrane of these cells, where it can be functional as an efflux transporter.

To confirm that the single-step clones of etoposide-selected MCF-7 cells, doxorubicin-selected IGROV-1 and doxorubicin-selected S-1 cells also showed an overexpression of ABCG2 at the protein level, western blots were performed on these sublines. The ABCG2 protein expression of the etoposide-selected MCF-7 clone 7 following normalisation to GAPDH levels was 1.9-fold greater than parental MCF-7 cells (Figure 2C). In addition, the IGROV-1 parental cells show no detectable expression of ABCG2 while clones 1–7 and 1–11, the two clones showing the greatest increase in *ABCG*2 mRNA expression, showed ABCG2 protein expression (data not shown).

Overexpression of *ABCG*2 *in vitro* can cause a mutation at position 482 (R482 → G or T), which alters the substrate specificity of this transporter (Honjo *et al*, 2001; Allen *et al*, 2002). To determine if this mutation was also present following low-dose, single-step selection, *ABCG*2 was sequenced following PCR with primers covering the region (aa 438–561) (see the Materials and Methods section in Supplementary Information). Several clones and MCF-7/ADR-VP3000 cells were sequenced. Sequence data showed that only the wild-type *ABCG*2 was present in all clones (data not shown), while the MCF-7/ADR-VP3000 cells had the mutation at position R482 → T (data not shown), consistent with what has been reported previously (Honjo *et al*, 2001).

To show the functional activity of the single-step doxorubicin-selected clones, we evaluated drug resistance. We examined the cytotoxicity of doxorubicin for the parental MCF-7 cells and the single-step-selected 14 nM clones and the 21 nM clone. The IC_50_ value for the parental cells was approximately 90 nM, while for the 14 nM doxorubicin-selected clones 1 and 2, the IC_50_ values were only 1.2-fold higher, indicating no increase in resistance to doxorubicin (Table 1). In contrast, the remaining 14 nM doxorubicin-selected MCF-7 clones demonstrated a 2- to 3.2-fold increase in resistance to doxorubicin. The 21 nM doxorubicin-selected clone was nearly four-fold more resistant to doxorubicin and over 90-fold more resistant to mitoxantrone (Table 1). Our data agree with previous reports that demonstrate more resistance towards mitoxantrone than doxorubicin in cell lines expressing the wild-type ABCG2 (Honjo *et al*, 2001). Thus, these clones show not only resistance to doxorubicin but also demonstrate MDR.

### ABCC4 does not confer resistance to doxorubicin

**Figure 3:**
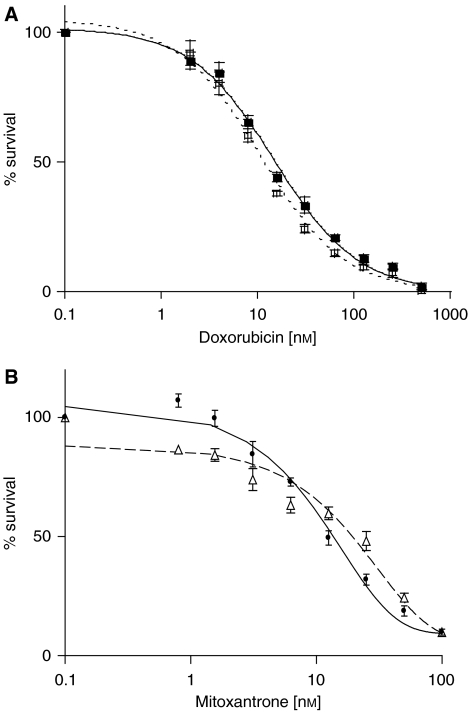
ABCC4 does not confer resistance to doxorubicin or mitoxantrone. (**A**) Cytotoxicity assays for ABCC4-overexpressing HEK293 (▪) and parental HEK293 (□) cells with doxorubicin. Dose–response curves were derived from three independent experiments using the CCK-8 assay. (**B)** Cytotoxicity assays for ABCC4-overexpressing HEK293 (▵) and parental HEK293 (•) cells with mitoxantrone. CCK-8 reagent was used for cytotoxicity assays as described in Materials and methods. Dose–response curves were derived from three independent experiments. In both (**A**) and (**B**), error bars indicate s.d. (*n*=3).

To determine if the expression of *ABCC*4 can confer resistance to either doxorubicin or mitoxantrone, additional studies using ABCC4-overexpressing HEK293 cells were performed. We previously demonstrated that only ABCC4 is overexpressed in these cells (Wu *et al*, 2005). Cytotoxicity assays with doxorubicin were performed comparing HEK293 cells to ABCC4-overexpressing HEK293 cells, and no significant differences were found in the ABCC4-overexpressing HEK293 compared to the parental HEK293 cells, IC_50_ values 14.9±1.2 and 10.7±1.0 nm, respectively (Figure 3A). Additional cytotoxicity assays were performed to determine if ABCC4 can confer resistance to mitoxantrone (Figure 3B). Similar to the assays with doxorubicin, no significant differences were found in the ABCC4-overexpressing HEK293 compared to the parental HEK293 cells. This suggests that ABCC4 is not responsible for the observed resistance to doxorubicin or mitoxantrone in single-step-selected clones.

### ABCG2 confers drug resistance to single-step doxorubicin-selected MCF-7 cells

**Figure 4:**
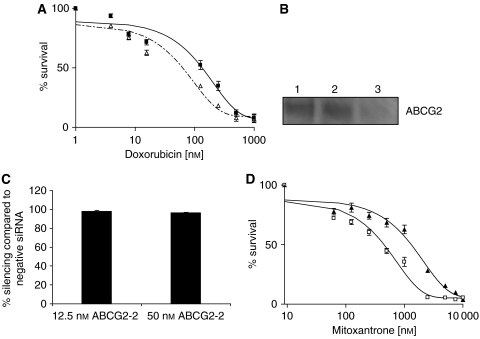
ABCG2 confers resistance to 21 nM single-step doxorubicin-selected clone. (**A**) Cytotoxicity assays using doxorubicin to evaluate the effect of inhibiting ABCG2 in the 21 nM single-step clone with 5 μM FTC. Dose–response curves were derived from six independent experiments using the CCK-8 assay for 21 nM cells with 5 μM FTC (▵) and without 5 μM FTC (▪). The mean values from six independent experiments are shown with error bars as s.e.m. (**B**) Western blotting analysis of ABCG2 protein using BXP-21 antibody following no treatment (lane 1), 50 nM negative siRNA treatment (lane 2) and 50 nM G2-2 siRNA treatment (lane 3). (**C**) Examination of two concentrations of siG2-2 siRNA on silencing of *ABCG2*. Levels of *ABCG2* following siRNA treatment were analysed using the QuantiGene Reagent System (Panomics). Levels were normalised to cyclophilin B (*PPIB*) mRNA and results reflect the average and s.d. (*n*=5). (**D**) Cytotoxicity assays using mitoxantrone to evaluate the effect of silencing *ABCG2* in the 21 nM single-step clone. Dose–response curves were derived from six independent experiments using the CCK-8 assay for 21 nM cells with 12.5 nM siG2-2 siRNA (□) and 21 nM cells with 12.5 nM siNeg (▴). The mean values from six independent experiments are shown with error bars as s.e.m.

To further evaluate if ABCG2 was conferring resistance to doxorubicin in the 21 nM MCF-7 clone, we performed cytotoxicity assays in the presence and absence of fumitremorgin C (FTC) (Rabindran *et al*, 2000), a specific inhibitor of ABCG2. With the addition of 5 *μ*M FTC, we were able to reverse the ABCG2-mediated resistance to doxorubicin (Figure 4A) to levels comparable to those of the parental MCF-7 cells (Table 1). The addition of FTC resulted in a 2.1-fold enhancement of the toxicity of doxorubicin on the 21 nM doxorubicin-selected MCF-7 cells. This indicates that the inhibition of ABCG2 results in greater toxicity of doxorubicin in these cells.

To confirm that ABCG2 was the ABC transporter conferring resistance to the single-step clones, RNA interference (RNAi) studies were also performed. Standard characterisation analysis was conducted to determine the degree of silencing mediated by a synthetic siRNA corresponding to ABCG2 (siG2-2). Using 50 nM siRNA, *ABCG2* mRNA was decreased by nearly 40-fold compared to a negative siRNA in the 21 nM MCF7 clone and ABCG2 protein expression was almost completely suppressed (Figure 4B). A lower concentration of siRNA was also examined, and comparable silencing was also observed at an mRNA level using 12.5 nM siRNA (Figure 4C).

Following validation, cytotoxicity assays were performed on cells treated with siG2-2 or negative control siRNA. The siG2-2 siRNA induced a shift of the drug response curve to the left, indicating less resistance to mitoxantrone (Figure 4D). The IC_50_ value for the siG2-2-treated cells was three-fold less than that for the negative siRNA-treated cells. Silencing with siG2-2 restored sensitivity to approximately 80–85% of the parental levels. Together with the results from the FTC cytotoxicity studies, this suggests that ABCG2 is critical for the drug resistance in the single-step 21 nM doxorubicin-selected MCF-7 cells.

### Histone acetylation is modified at the *ABCG*2 locus in the single-step doxorubicin-selected clones

**Figure 5:**
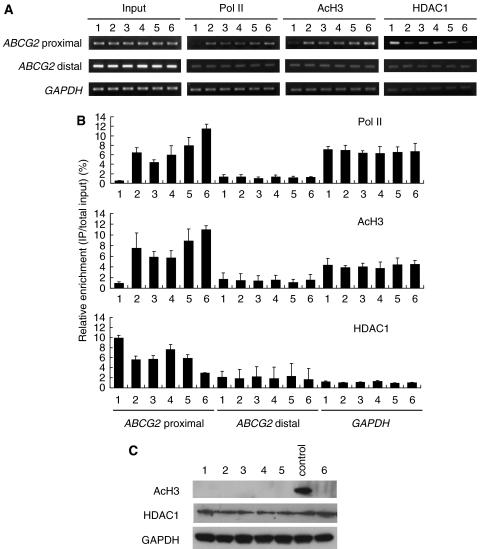
*ABCG2* chromatin in the doxorubicin-selected clones is associated with more RNA Pol II and AcH3 but less HDAC1 than the parental MCF-7 cell line. (**A**) Chromatin immunoprecipitation assays were performed with parental MCF-7 cells and the doxorubicin-selected clones (Kuo and Allis, 1999). A drug-resistant subline MCF7/FLV1000 (Robey *et al*, 2001) was employed as a control. Soluble chromatin used in immunoprecipitations had a typical size of <0.5 kb visualisation by gel electrophoresis. RNA Pol II, AcH3 or HDAC1 associated with the distal and proximal region in the *ABCG2* promoter were analysed by PCR. Lane 1, parental MCF-7; lane 2, 14 nM clone 2; lane 3, 14 nM clone 6; lane 4, 14 nM clone 13; lane 5, 21 nM clone; and lane 6, MCF7/FLV1000. Input: DNA isolated from the lysate before immunoprecipitation. A representative result from three independent experiments is shown. (**B**) Quantitative analyses of the occupancy of Pol II, AcH3 or HDAC1 to the *ABCG2* promoter (proximal and distal regions) in the cells. The results are expressed as the percentage of immunoprecipitate (IP) over total input DNA. Error bars show the s.d. of three independent experiments. Lanes 1–6 are the same as above. (**C**) Western blot analysis of AcH3 and HDAC1. Whole-cell lysates were prepared from the parental MCF-7 cells and the various single-step doxorubicin-selected MCF-7 clones for AcH3 (17 kDa) and HDAC1 (65 kDa) detection, respectively. Samples 1–6 are as follows: 1, parental MCF-7; 2, 14 nM clone 2; 3, 14 nM clone 6; 4, 14 nM clone 13; 5, 21 nM clone; and 6, MCF7/FLV1000. As a control, HDAC inhibition by depsipeptide results in hyperacetylation of H3 in the whole-cell lysate (control lane). Glyceraldehyde-3-phosphate dehydrogenase was used as a loading control for each sample.

To determine whether expression of ABCG2 was associated with epigenetic changes such as histone acetylation, we performed ChIP assays to measure the interaction of the protein with a specific DNA sequence *in vivo* (Kuo and Allis, 1999). We used ChIP to investigate the level of AcH3, HDAC1 and RNA Pol II associated with different segments of the *ABCG2* gene. Compared with the parental MCF-7 cells, the enrichment of H3 acetylation to the proximal *ABCG*2 promoter was enhanced more than 10-fold but there was less association of HDAC1 (approximately 50% decrease) in the selected clones and in a well-characterised resistant subline MCF7/FLV1000 (Robey *et al*, 2001) (Figure 5A and B). The loss of HDAC1 and the stronger binding of Pol II to the proximal promoter correlated well with the enhanced transcription of *ABCG*2 in these cells. As a control, the weak association of Pol II, AcH3 and HDAC1 to the distal *ABCG*2 promoter remained similar in the parental, resistant MCF7/FLV1000 and the doxorubicin-selected clones. The *GAPDH* promoter was employed as an additional control. Compared with the parental MCF-7 cells, the drug-selected clones expressed a similar level of *GAPDH*, as determined by RT–PCR (data not shown). To determine whether drug selection-related histone H3 hyperacetylation was specific to the *ABCG2* promoter, we assessed the association of Pol II, AcH3 and HDAC1 with the housekeeping gene *GAPDH* promoter. Accordingly, ChIP analysis did not reveal any appreciable difference in the binding of Pol II, AcH3 and HDAC1 to the *GAPDH* promoter between the parental and the drug-selected sublines (Figure 5A and B). This correlated with the strong binding of Pol II and AcH3 and the weak association of HDAC1 to the proximal promoter of *GAPDH* that did not change from the parental to the selected clones. These results demonstrate that increased histone H3 acetylation in the proximal *ABCG*2 promoter in response to doxorubicin selection was gene-specific.

In contrast, immunoblot analysis of whole-cell lysates demonstrated that drug selection did not globally change the level of histone H3 acetylation in the doxorubicin-selected clones or in MCF7/FLV1000 (Figure 5C). The lack of global changes in histone acetylation supports the notion that regional accumulation of acetylated histone (revealed by the ChIP assay) in the *ABCG*2 proximal promoter is specific to the *ABCG*2 promoter and specifically due to drug selection. Doxorubicin selection did not alter the levels of global core histone acetylation, which are undetectable. As a control, HDAC inhibition by depsipeptide results in hyperacetylation of H3 in the whole-cell lysate, again supporting the specificity of the reduced HDAC1 bound to the *ABCG*2 promoter (Figure 5C). The expression of HDAC1 was not significantly different among the various sublines.

## DISCUSSION

Doxorubicin is a chemotherapeutic agent principally used for the treatment of solid tumours, especially breast cancer and lymphoma (Minotti *et al*, 2004). Although doxorubicin can work through various mechanisms, it is still not immune to the MDR phenotype and, consequently, the development of resistance to doxorubicin has been well documented in a broad range of cell lines (Shen *et al*, 1986; Barrand *et al*, 1994; Mehta, 1994). To more closely examine the regulation of ABC transporter expression at concentrations similar to those *in vivo*, we established several single-step doxorubicin-selected clones with three different cancer cell lines and found that these clones did not express *ABCB*1 (Figure 1). Although ABCC2 and ABCC4 were overexpressed at the mRNA level, ABCG2 was the only transporter responsible for resistance in these clones (Figures 3 and 4). In addition, a podophyllotoxin derivative, etoposide (Hande, 1998), was also able to solicit the same response in MCF-7 cells after single-step selection (Figures 1B and 2C). Moreover, the selection of IGROV-1 and S-1 cancer cells with 14 or 21 nM doxorubicin also resulted in the overexpression of ABCG2 (Figure 1C and D). We cannot completely rule out the possibility that a pre-existing cell with high expression of *ABCG*2 was present in the parental population; nevertheless, we found an overexpression of *ABCG*2 following low-dose selection in three independent cell lines originating from different tissues using two different drugs. In addition, the lack of enrichment in the putative mammary cancer stem cell surface phenotype, CD44+/CD24− (Al-Hajj *et al*, 2003) (Supplementary Figure 1), in the single-step doxorubicin-selected MCF-7 clones further supports the theory of adaptation over selection with these studies.

Doxorubicin and etoposide are structurally different but they are both topoisomerase II inhibitors and this may explain the similarities in the response of cells to selection with these agents; however, further studies will be required to establish this. Others have also investigated the effects of single-step doxorubicin selection on the human sarcoma cell line MES-SA and have reported an increase in *ABCB*1 expression (Chen *et al*, 1994; Beketic-Oreskovic *et al*, 1995); yet previous studies were performed before the identification of ABCG2 as a major ABC transporter in the area of MDR. In addition, the authors used 40 nM doxorubicin for 14 days, nearly two-fold higher than the concentration we employed. We have examined the endogenous levels of *ABCG*2 in the MES-SA parental cells, and it is not detectable by real-time PCR (data not shown). It is possible that in this cell type, ABCB1 is responsible for resistance; however, as these original studies did not report other ABC transporters, we cannot address this question. We will determine in the future if a single-step drug regimen results in the same ABC transporters being overexpressed *in vivo*.

ABCG2 is a half-transporter that appears to have a protective role in a variety of stem cells to maintain progenitor cells in an undifferentiated state (Zhou *et al*, 2001) and is often referred to as a stem cell marker. This ABC transporter is also highly expressed in the placenta, liver and small intestine (reviewed by Krishnamurthy and Schuetz, 2006) where it is influential in the absorption and distribution of xenobiotics. However, to date, the role of this transporter in clinical drug resistance has not been evaluated in any extensive clinical trial (Robey *et al*, 2007). Interestingly, one smaller study with 59 primary breast cancer patients reported a negative correlation between *ABCG*2 mRNA expression and response rate and progression-free survival with anthracycline-based chemotherapy (Burger *et al*, 2003). Investigators first determined that stem cells from rhesus monkey bone marrow, mouse skeletal muscle and murine embryonic stem cells, which constitute the ‘side population’ of effluxing cells, expressed ABCG2 (Zhou *et al*, 2001). In addition, the expression of ABCG2 has been linked to prostate stem cells and mammary gland stem cells (Clayton *et al*, 2004; Huss *et al*, 2005). ABCG2 may serve as the primary line of defence against the cytotoxic effects of drug in our single-step-selected clones, as it does in stem cells. However, these cells were not enriched in the putative mammary cancer stem cell surface phenotype, CD44+/CD24− (Al-Hajj *et al*, 2003) (Supplementary Figure 1), suggesting that we have not selected for cancer stem cells in these studies and that the epigenetic changes that occurred in the sublines were a result of adaptation as opposed to selection. Others have also reported adaptation of cells to drug pressure as the mechanism of drug resistance (Matsumoto *et al*, 1997). Microarray analysis of these single-step doxorubicin-selected MCF-7 clones shows that gene expression changes were found primarily in molecular and cellular functions related to cell death, cancer and inflammatory disease (data not shown). This suggests that adaptation to cell stressors such as these may be responsible for turning on the drug resistance genes.

The inhibition of ABCG2 by FTC and the silencing of *ABCG2* through siRNA-mediated RNAi showed reversal of drug resistance, confirming that *ABCG2* overexpression is responsible for drug resistance (Figure 4). Although ABCC4 expression was seen in these clones, mitoxantrone was not a substrate for ABCC4 (Figure 3B), and ABCC4 did not confer resistance to doxorubicin (Figure 3A). Thus, this transporter is unlikely to be associated with drug resistance in the single-step-selected clones. Using PAGen@UIC (www.uic.edu/pharmacy/depts/pmpcpd/pagen/), we found that there are 11 predicted transcription factors that have binding sites on the promoters of both ABCG2 and ABCC4 (Kamalakaran *et al*, 2005). Thus, it is possible that the coordinate overexpression of both transporters may be due to an effect of common transcription factors. Further work is necessary to unravel the mechanism of this coordinate overexpression.

In this report, we have shown a reproducible pattern of histone acetylation at the *ABCG*2 gene that was modified in the clones selected by doxorubicin treatment and in resistant cells (MCF7/FLV1000) overexpressing *ABCG*2 (Figure 5). Presumably, HDAC1, specifically bound to the *ABCG*2 proximal promoter close to the transcriptional initiation site in the parental MCF-7 cells, suppressed histone acetylation at the *ABCG*2 gene. Therefore, as compared to the parental MCF-7 cells, the weaker association of HDAC1 with the proximal *ABCG*2 promoter in the selected clones and in MCF7/FLV1000 facilitated histone hyperacetylation and subsequently transcriptional upregulation of *ABCG*2 (Figure 5). Upregulation of *ABCB*1, a classical MDR gene, by chemotherapeutic drugs has also been shown to be associated with changes in the spatial and temporal patterns of histone H3 acetylation (Baker *et al*, 2005). Further interesting evidence was gleaned from our microarray mRNA expression studies coupled with pathway analysis, indicating that the oestrogen receptor may regulate the expression of *ABCG*2 (data not shown). This hypothesis is supported by recent work that showed the presence of an oestrogen response element in the *ABCG*2 gene (Ee *et al*, 2004). Additional studies are necessary to elucidate the role of the oestrogen receptor in *ABCG*2 overexpression. To our knowledge, this is the first report of *ABCG*2 overexpression following single-step selection with low concentrations of doxorubicin in breast, ovarian and colon cancer cells. Further work will elucidate the triggers that are required to alter expression of these transporters.

## Figures and Tables

**Table 1:** Sensitivity of clones to doxorubicin and mitoxantrone

	**Parental MCF-7**	**14 nM clone 1**	**14 nM clone 2**	**14 nM clone 6**	**14 nM clone 11**	**14 nM clone 13**	**14 nM clone 16**	**21 nM clone 1**
*Doxorubicin*								
IC_50_ (nM)	89.7	111.1	118.9	173.7	284.7	264.4	260.7	323.0
s.d.	28.2	27.3	50.8	46.3	56.7	65.6	51.8	63.4
								
*Mitoxantrone*								
IC_50_ (nM)	15.6	33.1	15.4	319.1	158.6	220.1	172.4	1407.0
s.d.	6.0	6.1	7.5	114.8	11.4	56.2	40.3	310.2

IC_50_ values are given in nM and are mean±s.d. The IC_50_ values were calculated from dose–response curves obtained from at least three independent experiments. Cytotoxicity assays were performed as described in Materials and Methods.
