# Supplementary material for: Single-step doxorubicin-selected cancer cells overexpress the ABCG2 drug transporter through epigenetic changes
Source: Br J Cancer. 2008 Apr 1;98(9):1515–24. doi: 10.1038/sj.bjc.6604334 (PMC2386965; doi:10.1038/sj.bjc.6604334)

**SUPPLEMENTAL DATA**

**Single-Step Doxorubicin-Selected Cancer Cells Overexpress the ABCG2 Drug Transporter through Epigenetic Changes**

Anna Maria Calcagno^1^, Jennifer M. Fostel^2^, Kenneth K.W. To^3^, Crystal D. Salcido^4^, Scott E. Martin^5^, Katherine J. Chewning^1^, Chung-Pu Wu^1^, Lyuba Varticovski^4^, Susan Bates^3^, Natasha J. Caplen^5^, and Suresh V. Ambudkar ^1,*^

^1^Laboratory of Cell Biology, Center for Cancer Research, National Cancer Institute, NIH, DHHS, Bethesda, MD 20892, USA, ^2^Alpha-Gamma Technologies, Inc., Raleigh, NC 27609, USA, ^3^Cancer Therapeutics Branch, ^4^Laboratory of Human Carcinogenesis, and ^5^Gene Silencing Section, Genetics Branch, Center for Cancer Research, National Cancer Institute, NIH, DHHS, Bethesda, MD 20892, USA

**SUPPLEMENTAL MATERIALS AND METHODS**

**Chemicals**

Acetoxy-methyl ester of calcein (Calcein-AM) was purchased from Molecular Probes (Eugene, OR). Doxorubicin was purchased from Calbiochem (San Diego, CA). Etoposide was purchased from Sigma. RT-PCR reagents were purchased from Roche Applied Sciences (Indianapolis, IN). Dulbecco’s modified Eagle’s medium (DMEM), Iscove’s modified Dulbecco’s medium (IMDM), L-glutamine, and penicillin/streptomycin were obtained from Invitrogen (Carlsbad, CA). Cell Counting Kit-8 was purchased from Dojindo Molecular Technologies, Inc. (Gaithersburg, MD). The siRNAs employed were obtained from Qiagen Inc. (Germantown, MD). The ABCG2-specific antibody BXP-21 and 5D3 were obtained from Kamiya Biomedical (Seattle, WA) and eBiosciences (San Diego, CA), respectively. Antibodies for CD44 and CD24 were purchased from BD Biosciences (San Jose, CA).

**Quantitative RT-PCR**

Real-time quantitative RT-PCR was used to measure the mRNA expression levels of the selected ABC transporters (ABCB1, ABCC1, ABCC2, ABCC4 and ABCG2). The LightCycler RNA Master SYBR Green Kit and LightCycler 480 instrument (Roche Biochemicals, Indianapolis, IN) were utilized in these studies. Specific PCR primer sequences for all ABC transporters and the reference gene are listed in Supplemental Table 1 and were designed using the LightCycler Probe Design 2 software unless otherwise noted. All primer sets were tested prior to use in this work to ensure that only a single product of the correct size was amplified for all primer sets. All RT-PCR reactions for ABC transporters were performed on 100-300 ng total RNA with 250 nM specific primers in the LightCycler480 as per manufacturers’ instructions. Negative controls consisting of no-template (water) reaction mixtures were run with all reactions. Melting curves were determined for each primer set following all RT-PCR runs using the LightCycler 480 software. Crossing points for each transcript were determined using the 2^nd^ derivative maximum analysis with the arithmetic baseline adjustment. Crossing point values for each transporter were normalized to the respective crossing point values for reference gene plasma membrane calcium ATPase 4 (PMCA4) (Calcagno et al., 2006). Data are presented as a fold change in gene expression for the drug-resistant cell lines compared to the parental cells with the same amount of total RNA using the delta delta Ct method.

**Sequencing of *ABCG*2 Position 482**

LightCycler Probe Design 2 software from Roche was used to design primers to amplify the sequence corresponding to amino acid position 482. RNA from several clones and from MCF-7/ADR-VP cells, which contain the mutation at amino acid 482, was amplified by RT-PCR using these primers: Forward primer, TTTCAGCAGTGTTT-CAGCCGTG and reverse primer, CAATGGTTGTGAGA

TTGACCAACAGAC. The DNA product for each sample was run on a 2% agarose gel then extracted using the QIAquick Gel Extraction kit by Qiagen. The samples were sequenced in both directions using an automated sequencer with the PRISM Big Dye Terminator Sequencing Kit (PerkinElmer Corp., Norwalk, CT).

**Surface Marker Analysis with Flow Cytometry**

Cells were washed with PBS prior to trypsinization. Cells are transferred to a 15ml conical tube containing 8-10ml media and then centrifuged at 1000 rpm for 5 min. The supernatant was removed and the pellet resuspended in 5ml of fresh media. Cells were incubated at 37˚C for 30 min. Cells were counted and transferred to eppendorf tubes (1x10^6^ cells/sample). Following a wash with PBS/0.5% BSA, cells were centrifuged and the supernatant removed. The appropriate fluorochrome-congugated antibody was added with PBS/0.5% BSA on ice in the dark for 30 minutes. Cells were washed with PBS/0.5% BSA then 7-AAD was added to all tubes except for the unstained and single stained cells. Cells were then analyzed on the flow cytometer. If compensation was required, compensation beads were utilized by adding one drop of the positive control beads and 1 drop of the negative control beads to an eppendorf tube followed by antibody. This would be incubated for 30 min at room temp. prior to washing with PBS/0.5% BSA. The suspension would then be analyzed on the flow cytometer. Laser filters used were the following: for FITC— FL1; for PE— FL2; for 7-AAD— FL3; and for APC— FL5 (red laser).

**SUPPLEMENTAL RESULTS**

**MCF-7 Single-Step Clones are not enriched in Mammary Stem Cell Surface Markers**

To determine if the single-step selection process causes an enrichment in stem cells by eliminating drug sensitive cells, we evaluated the cell surface markers of these clones. Others have shown that CD44 positive and CD24 negative cells are markers for mammary stem cells (Al-Hajj et al., 2003). Thus, we evaluated these markers. We found that these single-step MCF-7 clones are not enriched in any mammary stem cell surface markers (**Suppl. Fig. 1**). This indicates that we are not selecting for stem cells during our selection process and that the cancer cells are undergoing epigenetic changes during our single-step selection.

**REFERENCES**

Al-Hajj, M., Wicha, M.S., Benito-Hernandez, A., Morrison, S.J. & Clarke, M.F. (2003). Prospective identification of tumorigenic breast cancer cells. *Proc Natl Acad Sci U S A*, **100:** 3983-8.

Calcagno, A.M., Chewning, K.J., Wu, C.P. & Ambudkar, S.V. (2006). Plasma membrane calcium ATPase (PMCA4): a housekeeper for RT-PCR relative quantification of polytopic membrane proteins. *BMC Mol Biol*, **7:** 29.

**Supplemental Table 1. List of oligonucleotide primer sequences for the ABC transporters and plasma membrane**

**calcium pump for quantitative real time**  RT-PCR reactions

| **ABC transporter** | **Position of primer** | **Forward oligo sequence** | **Reverse oligo sequence** |
| --- | --- | --- | --- |
| ABCB1 | 834-1086 | GCCTGGCAGCTGGAAGACAAATAC | ATGGCCAAAATCACAAGGGTTAGC |
| ABCC1 | 3809-3967 | TGTGTGGGCAACTGCATCG | GTTGGTTTCCATTTCAGATGACATCCG |
| ABCC2 | 872-1027 | AATCAGAGTCAAAGCCAAGATGCC | TAGCTTCAGTAGGAATGATTTCAGGAGCAC |
| ABCC4 | 4818-4968 | GAGTTGCATGACTTGGACACGGTA | AACAGAGGGTTAGCCTTCCATAAATGAGA |
| ABCG2 | 266-646 | CCGCGACAGCTTCCAATGACCT | GCCGAAGAGCTGCTGAGAACTGTA |
| PMCA4 | 2309-2465 | ATCTGCATAGCTTACCGGGACT | TGCCAGCTTGTTTGCATTTGGCAATA |

**SUPPLEMENTAL FIGURE LEGENDS**

**Suppl. Figure 1. Single-Step Clones are not enriched in mammary stem cell markers.** Flow cytometric analysis of CD44 and CD24 on MCF-7 parental cells and the single-step 21 nM doxorubicin-selected clone. There is no enrichment of CD44+/CD24- cells for the single-step selected clone. Table shows the average of N=4. The histogram from a representative experiment (n=4) is shown.

**Suppl. Fig. 1**
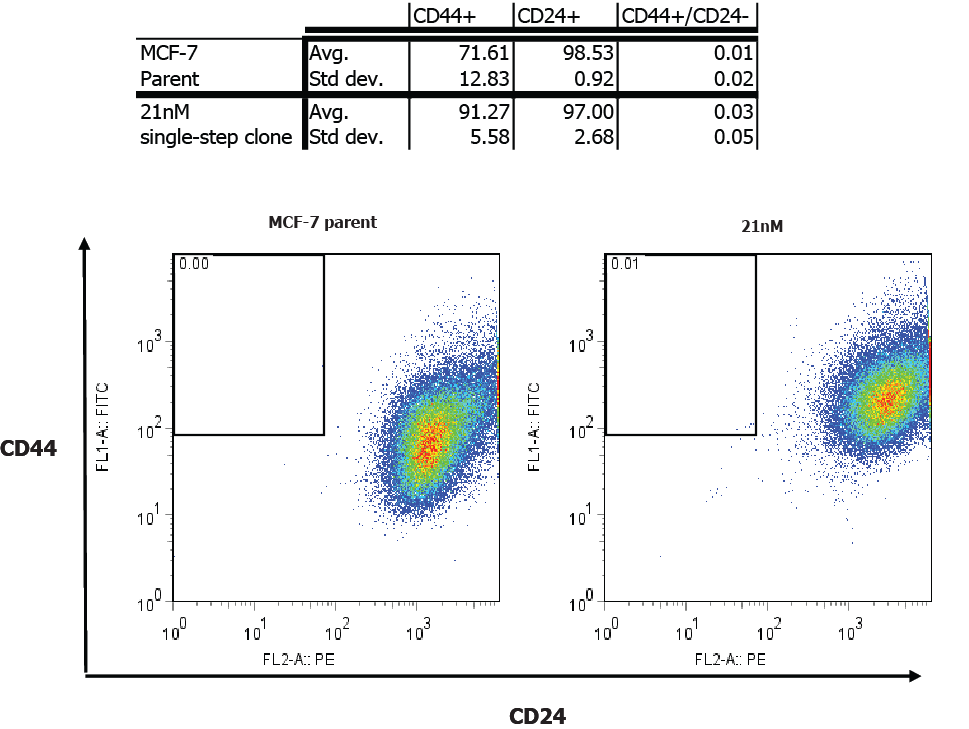

Supplement: Supplementary Data [file 6604334x1.doc]
